# Study on the Influence of Temperature and Water Content on the Static Mechanical Properties of Sandstone

**DOI:** 10.3390/ma17143399

**Published:** 2024-07-09

**Authors:** Xiaojun Zhang, Maolin He, Zhuo Li, Yongsheng Jia, Wenxue Gao

**Affiliations:** 1 Hubei Key Laboratory of Blasting Engineering of Jianghan University, Wuhan 430056, China; jason03566@163.com; 2 Faculty of Architecture, Civil and Transportation Engineering, Beijing University of Technology, Beijing 100124, China

**Keywords:** uniaxial compressive strength, moisture content, low temperature, elastic modulus

## Abstract

The area of permafrost worldwide accounts for approximately 20% to 25% of land area. In cold-climate regions of China, which are garnering international attention, the study of low-temperature and moisture effects on rock mass mechanical properties is of significant importance. China has a wide area of cold regions. This research can provide a foundation for China’s exploration activities in such extreme environments. This paper examines the mechanical behavior of rock specimens subjected to various low temperatures and water contents through uniaxial compression tests. The analysis encompasses failure modes, stress–strain relationships, uniaxial compressive strength (UCS), and elastic modulus (EM) of these specimens. Findings reveal that at lower temperatures, the rock specimens’ fracture patterns transition from compressive shear failure to cleavage failure, reflecting a shift from a plastic–elastic–plastic to a plastic–elastic response. Specifically, saturated rocks exhibit a 40.8% decrease in UCS and an 11.4% reduction in EM compared to their dry counterparts. Additionally, in cold conditions, an increased water content in rocks primarily leads to vertical cracking. Under such conditions, saturated rocks show a 52.3% decline in UCS and a 15.2% reduction in EM, relative to their dry state.

## 1. Introduction

The area of permafrost worldwide accounts for approximately 20% to 25% of land area. The global permafrost is mainly distributed in the northern hemisphere, especially in high-altitude areas such as Russia and Canada, as well as China, such as the Qinghai Tibet Plateau. The area of cold regions in China accounts for about 75% of the national territory. There are many cold regions in China, which are famous around the world [1].

Frozen rock and frozen soil changes are global issues. As a major country with permafrost and frozen rock, the study of China’s frozen rock is not only a necessity for scientific exploration, but also a necessary measure to address the challenges of climate change and ensure infrastructure security. It is also a necessary path to maintain ecological balance and sustainable socio-economic development. The study of the characteristics of frozen rocks in China is of great significance.

As part of its ongoing initiatives under the “Belt and Road” and “Western Development” strategies, China has been instrumental in initiating a multitude of projects in the colder regions such as Xinjiang and Xizang. These efforts, consistent with national policies [2,3], have led to significant development activities in these areas [4]. However, in some areas, the rock masses are constantly frozen. Due to the complexity of the geological environment, there are obvious differences in the internal water content of rocks during diagenesis. Variations in water content and levels of saturation significantly influence the physical and mechanical characteristics of rock masses in cold regions [5,6,7,8]. Furthermore, rock masses in these frigid areas are exposed to not only static loads but also dynamic influences such as blasting and mechanical construction activities [9,10]. Consequently, an in-depth analysis of the static mechanical properties of frozen rocks under different temperatures and saturation levels is vital for the effective management of rock mass stability in these cold environments. During tunnel excavation and blasting construction in these cold regions, it is possible to have a better understanding of rock characteristics and avoid unnecessary geological hazards.

Previous research endeavors have delved into the mechanical behaviors of rock specimens at low temperatures, with a focus on the mechanical attributes of frozen rock. These studies have primarily utilized methods such as indoor uniaxial compression, shear resistance evaluation, and Brazilian splitting tests to investigate these properties [11,12,13]. In particular, the article of Wang Chao [14] has centered on the analysis of residual deformation. This research involved subjecting a soil–rock mixture to multiple cycles of freezing and thawing. Findings from this research indicate that during freeze–thaw cycles, the soil–rock mixture undergoes repeated processes of frost heaving and thaw-induced contraction. It was observed that the initial instances of frost heave and thaw shrinkage exhibited significant changes. However, as the number of freeze–thaw cycles increased, a decrease in residual deformation was noted. Finally, the residual deformation tends to stabilize. Lv Zhitao [15] conducted a frost heave test on saturated sandstone, which exhibited open cracks. Investigations have revealed that in environments where freezing occurs uniformly, the phenomenon of frost heave in the fissures of highly permeable sandstone is more significant when contrasted with scenarios of unidirectional freezing. The interplay between the freezing dynamics and the permeability characteristics of the rocks plays a pivotal role in influencing the alterations in the water content within these fissures. So, different permeable rocks will produce different crack and frost heave modes under different freezing conditions. Zhu Chuanqu [16] studied the strength characteristics and mechanism of coal rock interface freezing through indoor experiments. A comprehensive set of direct shear experiments was performed on frozen coal and rock specimens under varying conditions of temperature, moisture content, and normal stress. Experimental findings indicate that the moisture level significantly affects the strength at the junction where coal and rock are frozen together. A notable increase in this strength was observed with rising moisture levels. For instance, during experiments conducted at a temperature of −10 °C, the frozen rock’s strength notably enhanced from 75.46 KPa to 267.42 KPa when moisture levels were elevated from 3% to 9%. Additionally, in a separate research study, Wang Ting [17] explored the pressure-melting phenomenon in frozen sandstone. This was done by conducting uniaxial compression tests and concurrently observing alterations in the electrical resistance within the sandstone. The findings showed that during the microcrack compaction stage, the electrical resistance of saturated frozen rock drops rapidly with increasing strain. However, during the elastic deformation and microcrack propagation stages, the resistance of sandstone decreases more gradually, contrasting notably with dry rocks. Furthermore, Wang Tingting [18] undertook laboratory-based Brazilian splitting tests to assess the tensile characteristics of fractured rock masses in cold environments that have undergone freezing. The research findings suggest that frozen rock specimens display characteristic brittle failure properties. There is a gradual decline in the tensile strength of frozen rock as the dimensions of cracks, both in width and length, increase.

Research has been conducted on the impact of water content on rock mechanical properties. Uniaxial compression, tensile, and direct shear tests were performed on specimens with varying moisture contents to analyze water’s weakening effect on rock strength [19,20,21,22]. Zhou Kunyou [23] investigated the impact of water content on rock strength through a series of uniaxial compression and tensile tests. The results indicated that water content significantly reduces the mechanical strength of rocks, transitioning their failure mode from a mixed tensile–shear type to predominantly tensile. Li Bo [24] performed direct shear tests on granite and sandstone specimens, which exhibited serrated fracture cracks. The rock specimens were subjected to three moisture conditions: dry, surface wet, and saturated. Findings suggest that surface moisture only alters the basic friction angle, while saturation leads to a reduction in both the unconfined compressive strength and basic friction angle of the rock specimens. Li Diyuan [25] established two parameters, namely the change in critical saturation and saturation per unit length, to analyze the rate of decline in rock strength and describe the distribution of water in cylindrical specimens. Using these two parameters, an analytical model was developed for calculating the normalized unconfined compressive strength of rocks with varying saturation levels. This model was applied to a tunnel project located in a fault zone in Yunnan as a case study. Kang Yongshui [26] focused on the mechanical properties of rock joints with a high content of clay. The findings from these experiments highlighted that the moisture content in the clay filler considerably affects the shear strength of the rock joint. Research has indicated that beyond the plastic limit of moisture content, there exists a negative correlation between the level of water content and the shear strength observed in rock joints.

Currently, many scholars have studied the mechanical properties of frozen rocks. Some scholars have also studied the influence of water content on the mechanical properties of rocks. However, the mechanical properties of rocks in a frozen state under different water content conditions are rarely studied by anyone. In cold regions, the mechanical properties of rocks are related to both low temperature and water content. So, it is necessary to explore the synergistic effect of low temperature and water content on rocks. Especially at low temperatures, the influence of different water contents on the mechanical properties of rocks is important. The influence of different low temperatures on the mechanical properties of rocks is also important under certain moisture content conditions. This study aims to evaluate the mechanical behavior of rock specimens subjected to different temperature and moisture conditions through uniaxial compression tests. This evaluation includes an examination of various aspects such as failure morphology, stress–strain responses, UCS, EM, and additional alterations in the rock specimens. This paper focuses on the influence of water content and low temperature on the mechanical properties of rocks. Special attention should be paid to the failure modes of rocks with different water contents at low temperatures.

## 2. Rock Static Mechanics Test Plan

### 2.1. Preparation of Rock Specimens

**Figure 1:**
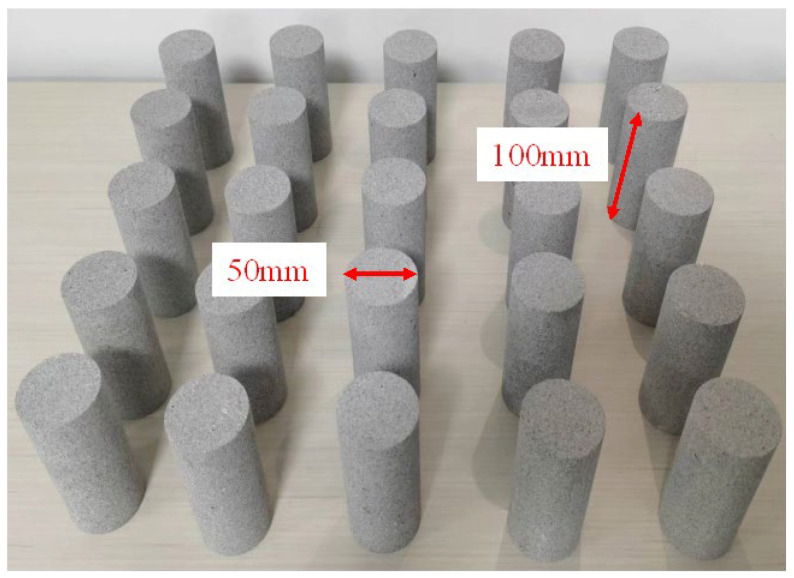
Rock specimens.

The sandstone specimens used in the experiment were taken from high-altitude areas in Sichuan. The main type of sandstone is Jurassic terrestrial sedimentary sandstone. These sandstones are mainly feldspar quartz sandstone and feldspar sandstone. The porosity of these sandstones is relatively low, generally below 12%. The specimen has a uniform texture and no obvious defects such as cracks or joints. The diameter of the specimen is 50 mm and the height is 100 mm. The nonparallelism and non-perpendicularity of each end face of the specimen are both less than 0.02 mm. The specimen size meets the basic requirements of the ISRM. To mitigate the effects of heterogeneity in rock specimens on experimental outcomes, an initial assessment of mass, volume, and longitudinal wave velocity was conducted for each specimen. Subsequently, specimens exhibiting significant discrepancies in these physical parameters were excluded from the test series. Representative rock specimens are depicted in Figure 1.

### 2.2. Rock Specimen Processing Schemes with Different Water Contents and Low Temperature

#### 2.2.1. Treatment Plan for Rock Specimens with Different Water Contents

All specimens were divided into a dry group, natural group, water absorption group, and saturated group. Each identical experiment was conducted three times. The results of the experiments were averaged.

1. The drying group specimens were dried in an electric drying oven at a temperature of 107 ± 1 °C for 48 h. The drying oven is shown in Figure 2.

**Figure 2:**
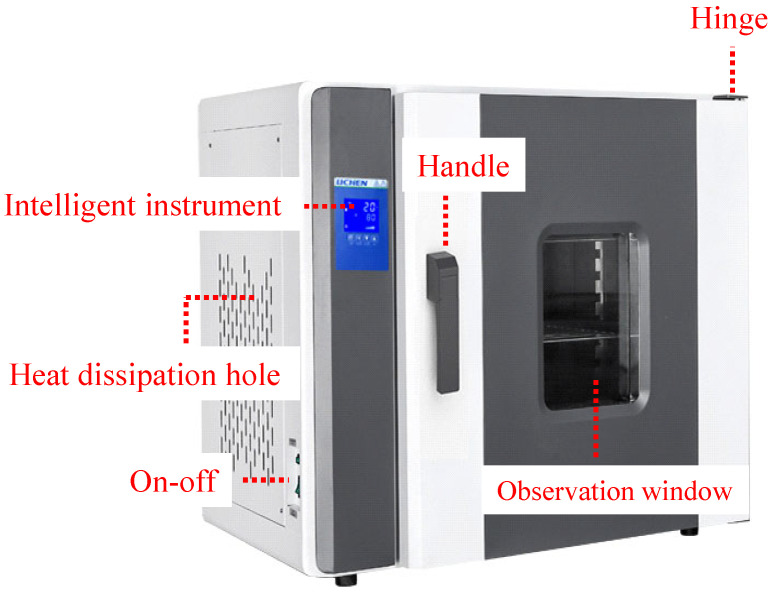
Drying oven.
2. The natural group specimens were first dried and then placed outdoors for 48 h, without any treatment.
3. The water absorption group specimens were first dried and then immersed in water step by step until submerged. Then, the rock specimens freely absorbed water for 48 h in the water.
4. The saturated specimens were dried first, and then gradually soaked in water until they were completely submerged. Then, the specimens absorbed water freely in water for 48 h. The saturation of the rock specimens was achieved using the boiling method. This process involved placing the specimens in a container of boiling water, ensuring that the water level remained consistently above the surface of the specimens throughout the procedure. The boiling time was not less than 6 h. The boiling bucket is shown in Figure 3.

The specimens post-drying were assumed to have a moisture content of zero. The moisture content in rock specimens can be determined by employing the following equation:

(1) $$w=\frac{m_{0}-m_{s}}{m_{s}}\times 100\%$$

where

*w*—Water content of rock specimens, %;
*m*_0_—Total mass of rock specimen after water absorption, g;
*m_s_*—Total mass of rock specimen after drying, g.

#### 2.2.2. Treatment Schemes for Rock Specimens at Different Low Temperatures

**Figure 4:**
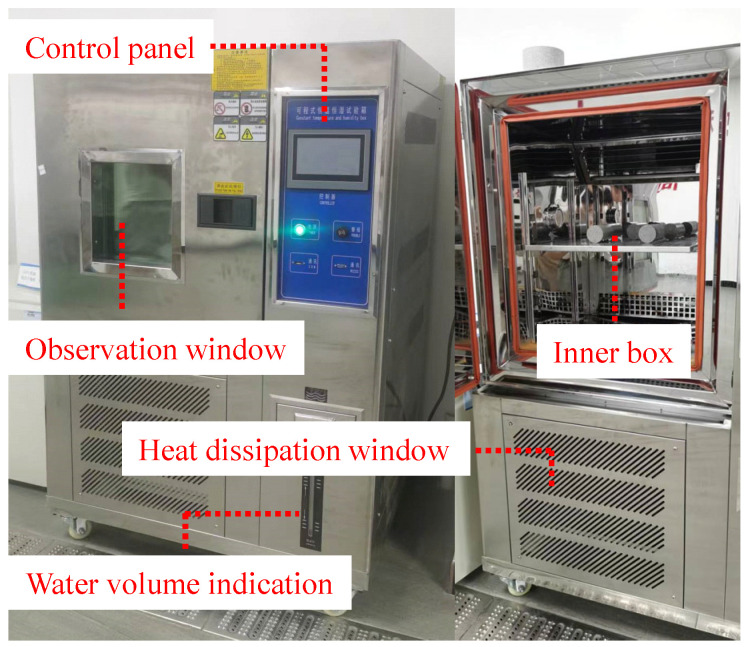
Constant temperature and humidity test chamber.

Rock specimens at different low temperatures were tested using a constant temperature and humidity test chamber, which is Jinheyuan brand. It is testing equipment used to simulate things or materials under different environmental conditions. It simulates different environmental conditions by controlling temperature and humidity for various tests and studies. When using a programmable constant temperature and humidity test chamber, users can set different temperature and humidity values as needed to simulate different environmental conditions. The constant temperature and humidity test chamber is shown in Figure 4.

This study primarily investigates the mechanical properties of rock specimens at various temperatures, specifically at 8 °C, 0 °C, −5 °C, −10 °C, −15 °C, and −20 °C.

To preserve the specific moisture content within each rock specimen with varying levels of water, every specimen was encased in plastic wrap prior to its placement in a chamber designed to maintain constant temperature and humidity. The specimens were then kept in this controlled environment for 24 h at a specific temperature set according to laboratory standards.

### 2.3. Rock UCS Test Plan

**Figure 5:**
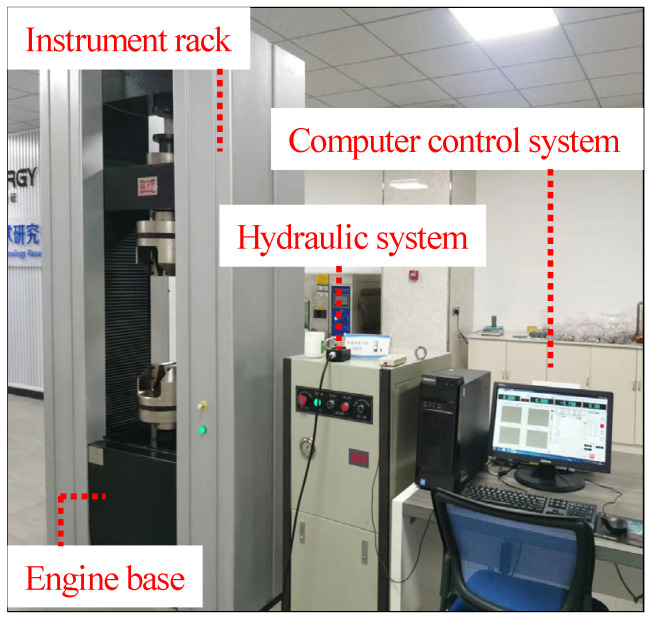
Electronic universal testing machine.

The static mechanical tests on rock specimens were conducted using a microcomputer-controlled electronic universal testing machine, model WDW-600E. This testing apparatus consists of a main unit, a drive motor coupled with a control system, a transmission mechanism, an integrated electronic machine for measurement and control, along with a computer equipped with data processing software and various supplementary components. The testing machine has a maximum force capacity of 600 kN. A depiction of this testing machine is provided in Figure 5.

The experiments discussed in this article primarily investigate the static mechanical properties of sandstone under various temperature conditions. These include analyzing the behavior of sandstone at both standard and low temperatures with different water content levels. The methodology for testing UCS in rocks was categorized into three separate groups, with a cumulative total of 42 experiments conducted. Each specimen was subjected to axial compression in a deformation-controlled manner until the rock specimen failed. The loading rate was 0.05 mm/min. Table 1 provides a detailed breakdown of these experimental groupings.

## 3. Test Results and Analysis

### 3.1. Water Content of Rock Specimens in Different States

After drying, placing outdoors, absorbing water, and boiling, the moisture content of the rock specimens was measured, and the results are shown in Table 2.

**Figure 6:**
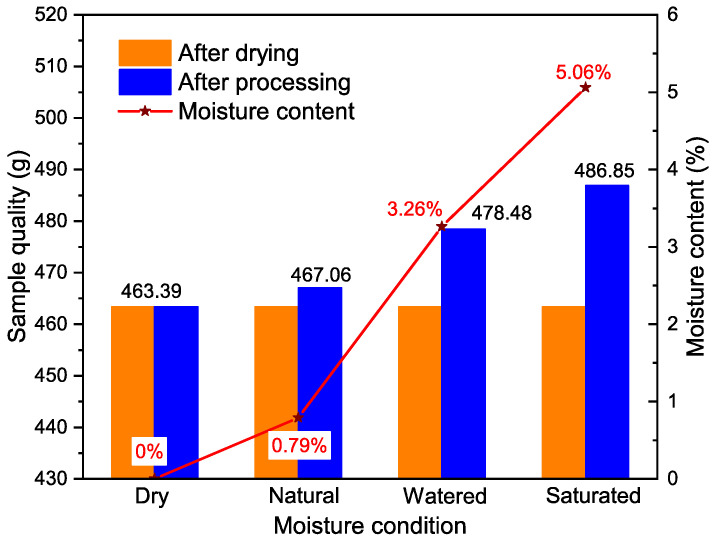
Water content of rocks in different states.

The data in the above table were plotted using professional drawing software Origin, as shown in Figure 6.

Observations from Figure 6 reveal that the water content in the rock specimens under natural conditions was measured at 0.79%. When the specimens reached a state of saturation, their water content increased to 5.06%. This change in water content illustrates the capacity of the rock to absorb water, thereby confirming the importance of examining the impact of water content on the mechanical properties of rocks.

### 3.2. Static Mechanical Properties of Sandstone under Different Low-Temperature Conditions

The specimens in their natural state (with a moisture content of 0.79%) were placed in conditions of ordinary temperature (8 °C), 0 °C, −5 °C, −10 °C, −15 °C, and −20 °C, respectively. The ordinary temperature was 8 °C as the reference temperature, which was determined according to the average temperature of Xizang in a year. Then, we observed the macroscopic failure characteristics and UCS characteristics of each specimen.

#### 3.2.1. Macroscopic Failure Characteristics of Rock Specimens

**Figure 7:**
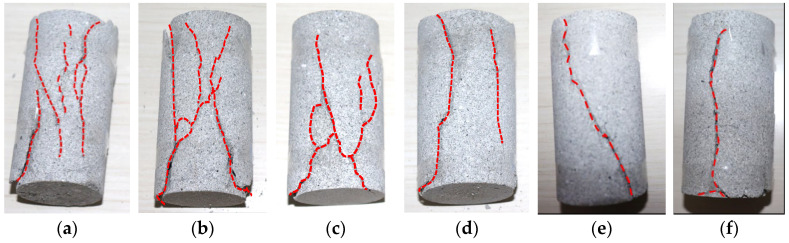
Failure morphology of rock specimens under different temperature conditions. (**a**) 8 °C; (**b**) 0 °C; (**c**) −5 °C; (**d**) −10 °C; (**e**) −15 °C; (**f**) −20 °C.

The failure morphology of the specimens under different low-temperature conditions is shown in Figure 7.

From Figure 7, it can be seen that under uniaxial compression at ordinary temperature (8 °C), the specimen exhibits complete failure and more cracks. It mainly manifests as compression shear failure. As the temperature decreases, the number of fracture cracks in rock specimens gradually decreases. At −20 °C, there is only one obvious crack in the specimen. It manifests as tensile failure (splitting failure).

#### 3.2.2. Static Strength Characteristics of Rock Specimens

**Figure 8:**
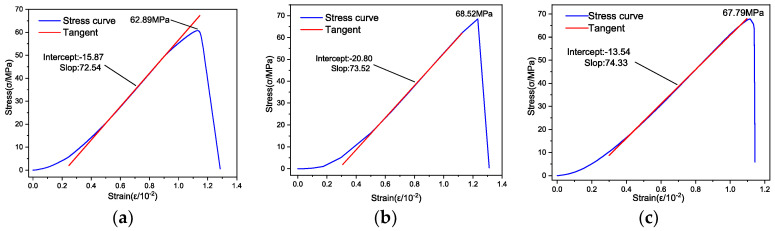

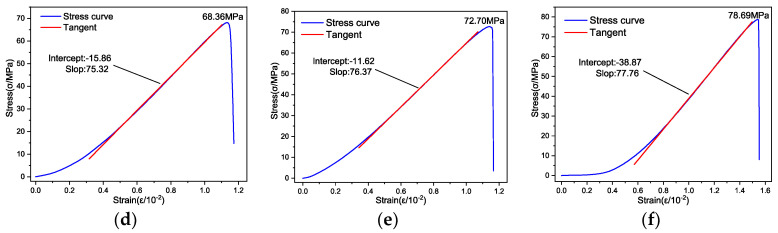
Stress–strain curves of the specimens under different temperature conditions. (**a**) 8 °C; (**b**) 0 °C; (**c**) −5 °C; (**d**) −10 °C; (**e**) −15 °C; (**f**) −20 °C.

The stress–strain curves of the specimens under different low-temperature conditions are shown in Figure 8.

Observations from Figure 8 indicate that at a standard temperature of 8 °C, the stress–strain curve initially exhibits an upward bend under low stress. As the stress reaches a certain threshold, the curve transitions into a linear trajectory. Eventually, it curves downward, forming a shape reminiscent of an “S”, continuing until the rock fractures. This behavior categorizes the rock specimen as a typical plastic–elastic–plastic body. When the temperature decreases, the shape of the stress–strain curve undergoes a noticeable alteration. Under low stress, the curve shows a slight upward deflection. As the stress intensifies, the curve becomes linear, maintaining this form up to the point of the rock’s destruction, indicative of a plastic–elastic nature of the rock specimen.

In scenarios where the water content of the specimens was at 0.79% (their natural state), Table 3 presents the values of compressive strength and EM at various temperatures. The calculation of the EM was done by identifying the linear segment of the stress–strain curve and using it as the reference tangent. The inclination of this tangent, highlighted by a red line in Figure 8, determines the specimen’s EM.

**Figure 9:**
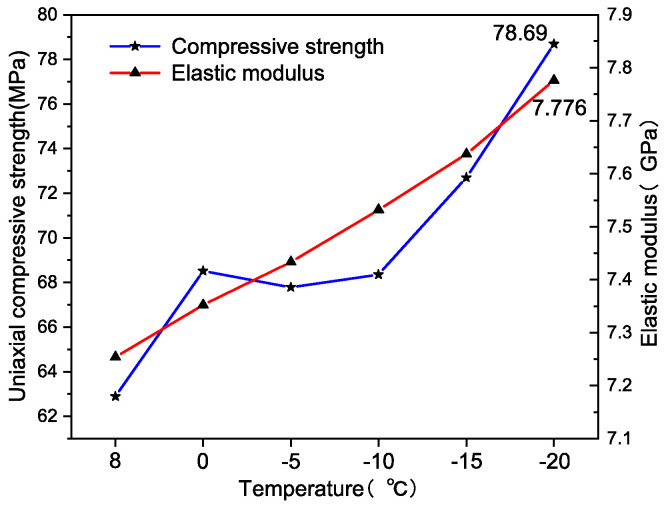
Trend of UCS and EM changes.

In order to analyze the variation in UCS and EM with temperature changes more intuitively, the data in Table 3 were plotted using professional Origin drawing software, as shown in Figure 9.

Upon examining Figure 9, it can be observed that both the UCS and the EM of rock specimens exhibit an increasing trend with decreasing temperature. Remarkably, when the temperature was lowered to −20 °C, the UCS of the rock specimen reached 78.69 MPa, while its EM was recorded at 7.776 GPa. It can be observed that at temperatures of 0 °C, −5 °C, and −10 °C, the UCS of the specimen tends to stabilize around 68 MPa. These data suggest that a reduction in temperature results in the specimens becoming harder and more brittle.

### 3.3. Static Mechanical Properties of Sandstone with Different Water Contents under Ordinary-Temperature Conditions

Rock specimens in different states, such as a dry state (moisture content of 0), natural state (moisture content of 0.79%), water absorption state (moisture content of 3.26%), and saturated state (moisture content of 5.06), were placed under ordinary temperature (8 °C) conditions. Then, we observed the macroscopic failure characteristics and static mechanical properties of the specimens.

#### 3.3.1. Macroscopic Failure Characteristics of Rock Specimens

**Figure 10:**
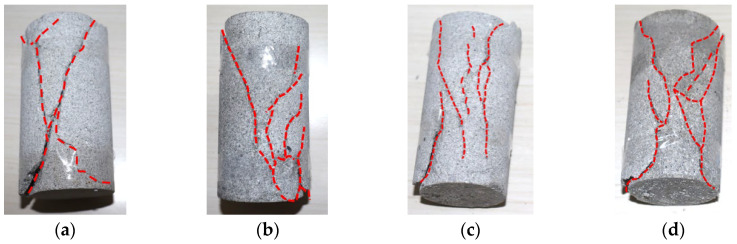
Failure morphology of rock specimens with different water contents. (**a**) Dry state (moisture content 0%); (**b**) Natural state (moisture content 0.79%); (**c**) Watered state (moisture content 3.26%); (**d**) Saturated state (moisture content 5.06%).

The macroscopic failure morphologies of specimens with different moisture contents at ordinary temperature (8 °C) are shown in Figure 10.

Observations from Figure 10 indicate that a rock specimen, when completely dry, with zero moisture content, exhibits fractures that are comparatively regular and fewer in number. With an increase in moisture levels, the incidence of cracking in the rock specimens becomes more pronounced. Moreover, the cracks of the rock specimen intersect with each other, presenting an X-shaped conjugate oblique shear failure. In a saturated state (with a water content of 5.06%), the crack propagation of the rock specimen is relatively obvious. Moreover, at the interface where the surface of the specimen intersects with the crack, some parts of the fractured rock mass fall off.

#### 3.3.2. Static Strength Characteristics of Rock Specimens

**Figure 11:**
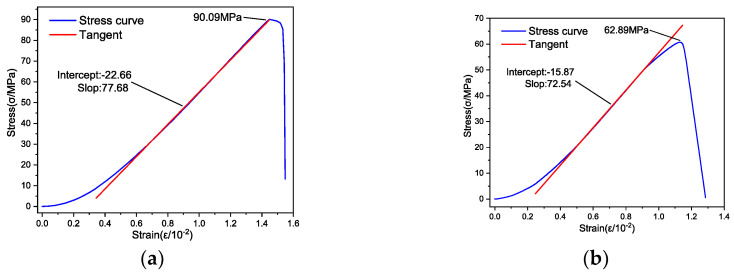

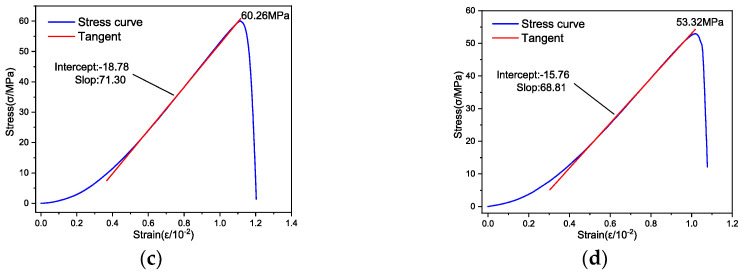
Stress–strain curves of rock specimens with different water contents. (**a**) Dry state (moisture content 0%); (**b**) Natural state (moisture content 0.79%); (**c**) Watered state (moisture content 3.26%); (**d**) Saturated state (moisture content 5.06%).

The stress–strain curves of rock specimens with different water contents at ordinary temperature (8 °C) are shown in Figure 11.

Observations from Figure 11 indicate that at an ordinary temperature of 8 °C, the stress–strain curves of rock specimens with varying moisture content levels initially bend upwards under low stress. As the stress intensifies to a certain level, these curves transition into a linear phase. Further increases in stress lead to a distinct behavior in the rock specimen in its natural state (with a moisture content of 0.79%), wherein its stress–strain curve bends downwards, ultimately forming an “S” shape. In contrast, the stress–strain curves of rock specimens with other moisture content levels remain linear until the point of destruction.

Table 4 presents the compressive strength and EM of rock specimens with different moisture levels at a standard temperature of 8 °C. The approach to calculate the EM is consistent with the method described earlier, as depicted by the red line segment in Figure 11.

**Figure 12:**
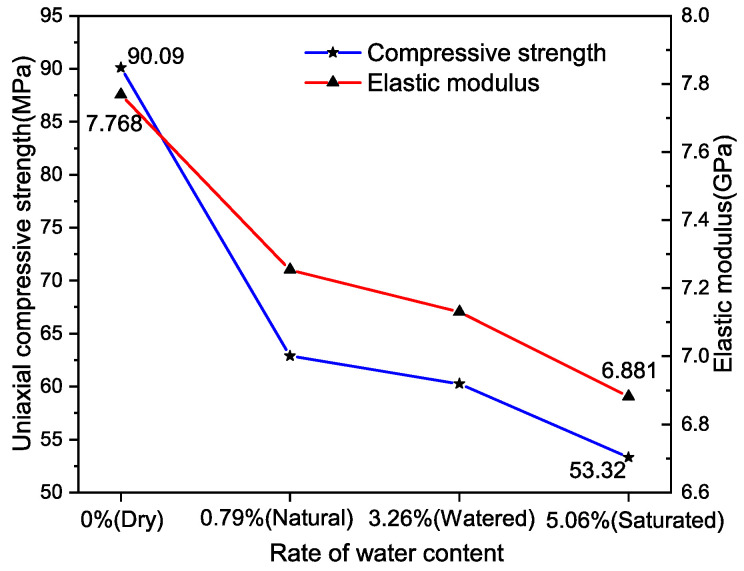
Trend of UCS and EM changes.

The information presented in Table 4 demonstrates a variation in the UCS of the rock specimens, ranging between 90.09 MPa and 53.32 MPa. This variation underscores the significant impact that moisture content has on the compressive strength of these specimens. The EM values for the specimens predominantly cluster around 7 GPa. For a more visual analysis of how UCS and EM correlate with water content, the information from Table 4 was graphically represented using advanced Origin drawing software, as demonstrated in Figure 12.

Analysis of Figure 12 reveals that both the UCS and the EM of the rock specimens exhibit a decline as the water content increases. In a dry state (moisture content of 0%), the UCS of a specimen was recorded at 90.09 MPa, and the EM was measured at 7.768 GPa. However, when a specimen was fully saturated (moisture content of 5.06%), there was a significant reduction in UCS to 53.32 MPa, amounting to a decrease of 40.8%. This observation indicates the profound impact of water on the strength of the rock. Simultaneously, the EM of the rock specimen experienced an 11.4% reduction, suggesting an increase in ductility.

### 3.4. Static Mechanical Properties of Sandstone with Different Water Contents under Low-Temperature Conditions

Rock specimens in different states, such as a dry state (moisture content of 0), natural state (moisture content of 0.79%), watered state (moisture content of 3.26%), and saturated state (moisture content of 5.06), were subjected to low-temperature (−5 °C) conditions. Then, we observed the macroscopic failure characteristics and static strength characteristics of the rock specimens.

#### 3.4.1. Macroscopic Failure Characteristics of Rock Specimens

**Figure 13:**
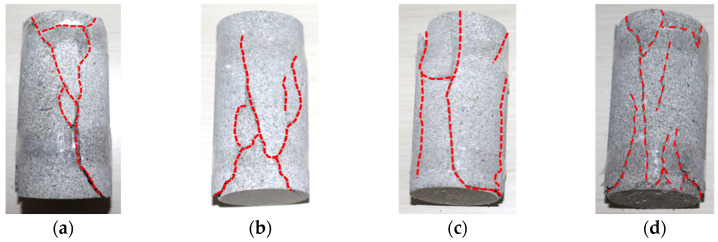
Failure morphology of rock specimens with different water contents. (**a**) Dry state (moisture content 0%); (**b**) Natural state (moisture content 0.79%); (**c**) Watered state (moisture content 3.26%); (**d**) Saturated state (moisture content 5.06%).

The macroscopic failure morphology of specimens with different moisture contents under low-temperature (−5 °C) conditions is shown in Figure 13.

From Figure 13, it can be seen that at low temperatures (−5 °C), as the water content increases, the number of cracks in the rock specimen increases. At the same time, in both dry and natural states, the crack direction is mainly inclined, and the rock specimens were mainly subjected to compression shear failure. In both water-absorbing and saturated states, the crack direction of the rock specimens is mainly vertical, while the rock specimens were mainly subjected to tensile failure.

#### 3.4.2. Static Strength Characteristics of Rock Specimens

**Figure 14:**
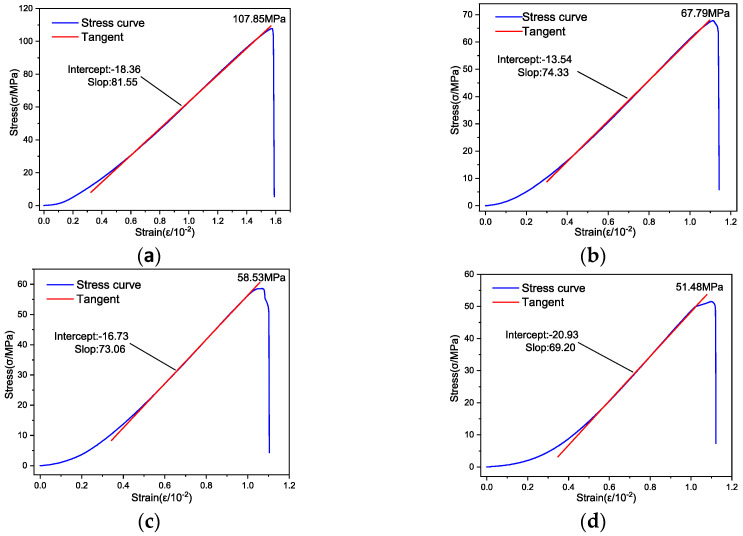
Stress–strain curves of rock specimens with different water contents. (**a**) Dry state (moisture content 0%); (**b**) Natural state (moisture content 0.79%); (**c**) Watered state (moisture content 3.26%); (**d**) Saturated state (moisture content 5.06%).

The stress–strain curves of rock specimens with different water contents under-low temperature (−5 °C) conditions are shown in Figure 14.

Figure 14 demonstrates that at a low temperature of −5 °C, the stress–strain curves of all rock specimens initially exhibit an upward bend under low stress. As the stress level increases, these curves become linear. Towards the end of the stress–strain relationship, the curves of specimens in both the dry state (moisture content of 0%) and the natural state (moisture content of 0.79%) remain linear up to the point of rock failure. Conversely, the curves for specimens in water-absorbing states (with a moisture content of 3.26%) and saturated states (with a moisture content of 5.06%) bend downwards until the destruction of the rock. This pattern suggests that with increasing water content, the behavior of the rock specimens transitions from a plastic–elastic nature to a more plastic–elastic–plastic characteristic.

Table 5 displays the compressive strength and EM of rock specimens with varying water content levels at a low temperature of −5 °C. The approach for calculating the EM was consistent with previously mentioned methods, as exemplified by the red line segment in Figure 14.

**Figure 15:**
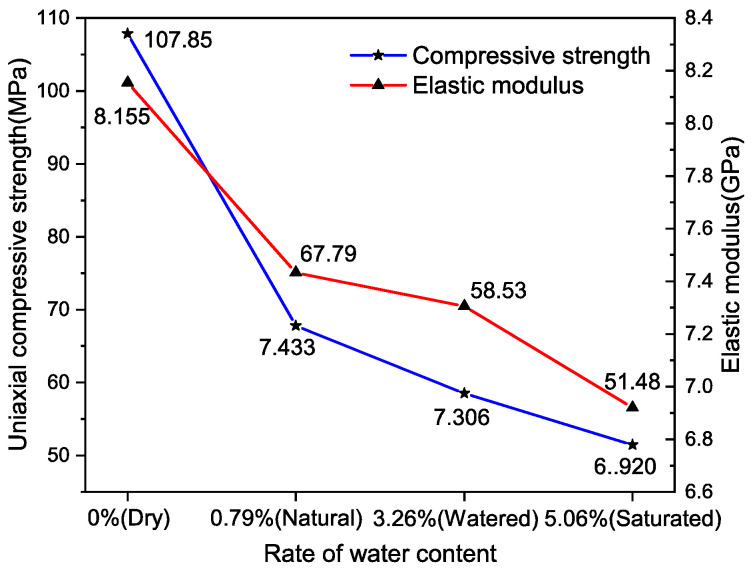
Trend of UCS and EM changes.

Data from Table 5 show that at a low temperature of −5 °C, the UCS of rock specimens varied significantly, ranging from 107.85 MPa to 51.48 MPa. This considerable range in UCS suggests that at low temperatures, water content significantly influences the strength characteristics of rocks. The EM of these rock specimens was observed to lie between 8.155 GPa and 6.920 GPa. For a more visual analysis of how the UCS and EM correlate with water content, the information from Table 5 was graphically represented using advanced Origin drawing software, as depicted in Figure 15.

Analysis of Figure 15 reveals that at a low temperature of −5 °C, both the UCS and the EM of rock specimens exhibit a decline as the water content increases. When the specimens were in a dry state (moisture content of 0%), the UCS was recorded at 107.85 MPa, and the EM was measured at 8.155 GPa. Conversely, in a saturated state (moisture content at 5.06%), the UCS decreased to 51.48 MPa, showing a reduction of 52.3%. Similarly, the EM dropped to 6.920 GPa, marking a decrease of 15.2% in comparison to the dry state. These observations underscore that under low-temperature conditions, the moisture content significantly impacts the strength and elastic properties of rock specimens.

## 4. Discussion

In this paper, we divided specimens into three groups for experiments and conducted a total of 42 experiments. All experimental results are listed in Table A1 in Appendix A. The scope of our experiments encompassed an investigation into the impact of low temperatures on the mechanical properties of rock specimens. Additionally, we examined how varying water contents affect these properties at an ordinary temperature of 8 °C, as well as at a low temperature of −5 °C. Each set of tests was analyzed to determine the macroscopic fracture characteristics and static strength characteristics of the rock specimens. Investigations into the influence of sub-zero temperatures and moisture levels on the structural integrity of rock specimens, particularly in terms of crack quantity and shape, remain notably scarce from other papers. This study’s findings hold considerable value for guiding construction projects in permafrost zones and hydrous rock formations, especially under conditions where fissures filled with water are prevalent in rocks in cold climates. The importance of acknowledging the decrease in the strength and ductility of rock masses under these circumstances cannot be overstated.

The study conducted by Zhao Yangchun [27] highlights a notable increase in the peak strength of frozen sandstone with a decrease in temperature, aligning with the outcomes presented in this research. Observations indicate a positive correlation between lower temperatures and the enhancement of UCS and EM in rock specimens. This strengthening and increased brittleness of rock specimens can be attributed to the solidification of water within their pores, resulting in a gradual augmentation of the EM of the pore ice. Consequently, this process elevates the overall relative EM of the rock. Furthermore, the amplification of strength in these frozen rocks can also be linked to the contraction of minerals present within the rocks, as noted in reference [28]. At the same time, Zhao Yangchun proposed, “With the temperature decreasing, the non-linear behavior of yield stage weakens”. This is consistent with our suggestion that as the temperature decreases, the rock specimen gradually transforms from a plastic–elastic–plastic body to a plastic–elastic body. This is due to the shrinkage and deformation of the rock skeleton caused by low temperatures, leading to the closure of some pores [29]. The rock becomes denser. The process of the rock yielding stage is shortened.

**Figure 16:**
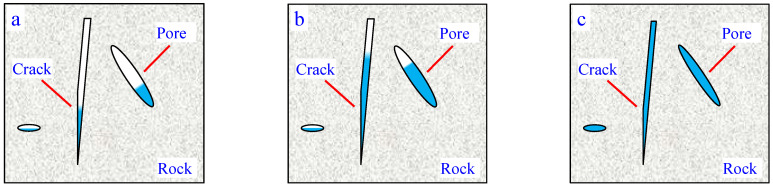
Schematic diagram of mechanical behavior of low-temperature rock specimens. (**a**) Ordinary temperature; (**b**) Lower temperature; (**c**) Low temperature.

Therefore, through analysis, we can know that the increase in uniaxial compressive strength and elastic modulus of rocks at low temperatures is mainly due to the solidification of water in the rocks at low temperatures. Due to the solidification of water, its volume expands and fills the pores in the rock, as shown in Figure 16. At the same time, the skeleton and particles of the rock may shrink at low temperatures [30]. The rock becomes denser at low temperatures, so its strength increases.

In our study, it was observed that both the uniaxial compressive strength (UCS) and the EM of the rock specimens exhibited a decline as the moisture content increased. This heightened level of moisture was found to adversely affect the strength of these rock specimens. At the ordinary temperature, the strength of rocks saturated with water was reduced by 40.8%, compared to dry rocks. This indicates that the presence of water can have a deteriorating effect on the mechanical properties and stability of rock masses. The gradual dissolution of the binding substances within the rock due to water exposure results in a diminished bonding force among the mineral particles of the rock [31]. This process ultimately leads to a reduction in the rock’s strength [32]. Tomor AK [33] noted in his research that there was a significant decrease in the UCS of sandstones when subjected to varying moisture levels. Specifically, it was observed that Darney stone lost 50% of its UCS under conditions of air dryness. The findings of Tomor AK corroborate the conclusions drawn in this study. However, on the basis of his research, this article also adds an analysis of the influence of water content on the mechanical properties of rocks at low temperatures. The study observed that the weakening effect of water on rock strength is amplified under low-temperature conditions, which can be attributed to the simultaneous presence of water, ice, and rock within the specimen at these temperatures [34]. Each component exhibits distinct temperature sensitivity, leading to uneven contraction of the rock specimens [35]. This differential response significantly influences the initiation and expansion of new microcracks [36].

**Figure 17:**
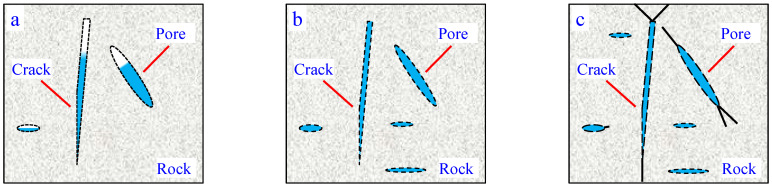
Schematic diagram of mechanical behavior of rock specimens with different water contents under low-temperature conditions. (**a**) Natural state; (**b**) Watered state; (**c**) Saturated state.

So, an increase in the water content of rocks will lead to a weakening of their strength. At an ordinary temperature, the water inside the rock will dissolve a portion of the cementing substance to weaken its bonding strength. At low temperatures, various substances inside rocks will contract or expand in different ways. Uneven shrinkage of rocks can lead to the expansion of existing cracks or the generation of new cracks. As shown in Figure 17, with the increase in water content inside the rock, the cement inside the rock is dissolved and the number of cracks inside the rock also increases.

This study assessed the mechanical behavior of rocks under varying low-temperature and moisture conditions. It was observed that lower temperatures generally enhance the mechanical robustness of rocks while simultaneously increasing their brittleness. Furthermore, the presence of water was found to reduce rock strength, an effect that was more pronounced in low-temperature environments. The insights and conclusions derived from this research offer valuable guidance for future studies on the dynamic mechanical properties of rocks in cold and moist conditions.

## 5. Conclusions

1. China has a large number of cold regions. It is meaningful to study the mechanical properties of rocks in cold regions. This article investigates the mechanical behavior of rock specimens under different low-temperature and water content conditions through uniaxial compression tests.
2. In natural conditions, rock specimens demonstrate a morphological transformation in fracture patterns with decreasing temperature, transitioning from compressive shear failure to a more distinct splitting failure. This shift signifies the rock’s transition from a combined plastic and elastic–plastic state to a predominantly plastic–elastic state. Moreover, the UCS and EM of the rocks exhibit a gradual increase as the temperature decreases.
3. Under standard temperature conditions, an elevation in the moisture content of rock specimens correlates with an increase in the number of failure cracks. A trend of decreasing UCS and EM was observed in the rocks as the water content rose. Compared to their dry state, where the moisture content was zero, the UCS of rocks in a saturated state experienced a reduction of 40.8%, and their EM decreased by 11.4%.
4. Under low-temperature conditions, as the water content of rock specimens increases, the direction of rock cracks is mainly vertical. The rock gradually transforms from a plastic–elastic body to a plastic–elastic–plastic body. The UCS and EM gradually decrease as the water content increases. Compared to the dry state, the UCS of saturated rocks decreased by 52.3%, and the EM decreased by 15.2%.
5. Overall, lower temperatures generally enhance the mechanical robustness of rocks while simultaneously increasing their brittleness. Furthermore, the presence of water was found to reduce rock strength, an effect that is more pronounced in low-temperature environments.
6. This study reveals the static mechanical properties of sandstone under low-temperature and different water content conditions. The research results can provide reference and guidance for future research on the dynamic mechanical properties of rocks under cold and humid conditions.

## Figures and Tables

**Figure 3:**
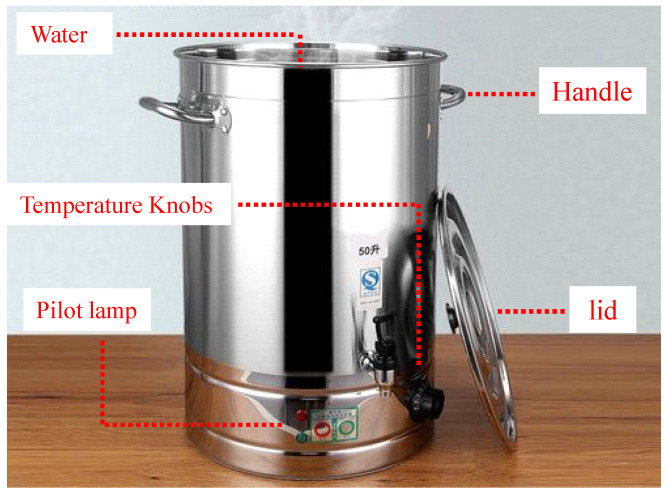
Boiling bucket.

**Table 1:** Grouping of UCS tests.

Groups	Moisture Condition	Temperature (°C)	Test Method	Number of Tests
T1-1	Natural	8	Uniaxial compression	3
T1-2		0		3
T1-3		−5		3
T1-4		−10		3
T1-5		−15		3
T1-6		−20		3
T2-1	Dry	8	Uniaxial compression	3
T2-2	Natural			3
T2-3	Watered			3
T2-4	Saturated			3
T3-1	Dry	−5	Uniaxial compression	3
T3-2	Natural			3
T3-3	Watered			3
T3-4	Saturated			3

**Table 2:** Water content of rock specimens in various states (unit: g).

Disposal Method	Dry	Natural	Watered	Saturated
Quality after drying	463.39	463.39	463.39	463.39
Quality after treatment	463.39	467.06	478.48	486.85
Moisture content	0	0.79%	3.26%	5.06%

**Table 3:** Statistical table of compressive strength and EM of specimens at different temperatures.

Temperature (°C)	8	0	−5	−10	−15	−20
UCS (MPa)	62.89	68.52	67.79	68.36	72.70	78.69
EM (GPa)	7.254	7.352	7.433	7.532	7.637	7.776

**Table 4:** Statistical table of UCS and EM of rock specimens under different water content conditions.

Moisture Content	Dry (0%)	Natural (0.79%)	Watered (3.26%)	Saturated (5.06%)
UCS (MPa)	90.09	62.89	60.26	53.32
EM (GPa)	7.768	7.254	7.130	6.881

**Table 5:** Statistical table of compressive strength and EM of rock specimens under different water content states.

Moisture Content	Dry (0%)	Natural (0.79%)	Watered (3.26%)	Saturated (5.06%)
UCS (MPa)	107.85	67.79	58.53	51.48
EM (GPa)	8.155	7.433	7.306	6.920

## Data Availability

The data used to support the findings of this study are included within the article.

## Nomenclature

°C	Celsius degree
*w*	Water content of rock specimens
*m*_0_	Total mass of rock specimen after water absorption
*ms*	Total mass of rock specimen after drying
UCS	Uniaxial compressive strength
EM	Elastic modulus
mm	Millimeter
g	Gram
min	Minute
MPa	Megapascal
GPa	Gigapascal

## Appendix A

**Table A1:** Uniaxial compressive strength test results.

Groups	Moisture Condition	Water Content	Temperature (°C)	Test Method	UCS (MPa)	EM (GPa)
T1-1	Natural	0.79%	8	Uniaxial compression	62.89	7.254
T1-2			0		68.52	7.352
T1-3			−5		67.79	7.433
T1-4			−10		68.36	7.532
T1-5			−15		72.70	7.637
T1-6			−20		78.69	7.776
T2-1	Dry	0	8	Uniaxial compression	90.09	7.768
T2-2	Natural	0.79%			62.89	7.254
T2-3	Watered	3.26%			60.26	7.130
T2-4	Saturated	5.06%			53.32	6.881
T3-1	Dry	0	−5	Uniaxial compression	107.85	8.155
T3-2	Natural	0.79%			67.79	7.433
T3-3	Watered	3.26%			58.53	7.306
T3-4	Saturated	5.06%			51.48	6.920
